# Pulmonary congenital cystic adenomatoid malformation, type I, presenting as a single cyst of the middle lobe in an adult: case report

**DOI:** 10.1186/1746-1596-2-17

**Published:** 2007-06-07

**Authors:** Luca Morelli, Irene Piscioli, Stefano Licci, Salvatore Donato, Alessia Catalucci, Franca Del Nonno

**Affiliations:** 1 Department of Pathology, "S. Maria del Carmine" Hospital, Rovereto (TN), Italy; 2 Department of Radiology, Civil Hospital of Budrio (BO), Italy; 3 Department of Pathology, "National Institute for Infectious Diseases – L. Spallanzani" IRCCS, Rome, Italy; 4 Department of Radiology, Civil Hospital of Bentivoglio (BO), Italy; 5 Department of Radiology, "S. Salvatore" Hospital, L'aquila, Italy; 6 Department of Pathology, Istituto Nazionale per le Malattie Infettive (INMI) "Lazzaro Spallanzani", IRCCS, Via Portuense, 292 00149 Roma, Italy

## Abstract

**Background:**

Congenital cystic adenomatoid malformation (CCAM) of the lung is an uncommon fetal development anomaly of the terminal respiratory structures. The large cyst type usually occurs in stillborn infants or newborn infants with respiratory distress. Cases of CCAM have been previously described in adulthood, more often type I with multiloculated cystic lesions.

**Case presentation:**

We report a case of type I CCAM presenting as a single, expansive cystic mass in the middle pulmonary lobe in a 38-year-old man, revealed by persistent cough and haemoptysis. Computed tomographic scan showed a single cyst with air fluid level, occupying the lateral segment of the lobe. When the type I CCAM is a single cyst, other cystic pulmonary lesions must be excluded. The intrapulmonary localization and the absence of cartilage in the cyst wall are conclusive findings of CCAM. The pathogenesis, management and differential diagnosis with other lung malformations are discussed along with a review of the literature.

**Conclusion:**

The literature data confirm that surgical resection is the treatment of choice in all cases of CCAM and in the cases of cystic pulmonary lesions with uncertain radiological findings, in order to perform a histological examination of the lesion and to prevent infection and the potential neoplastic transformation.

## Background

The development of the respiratory system begins at 3 weeks of gestation, and aberrations in developmental processes may give rise to a group of structural abnormalities collectively referred to as bronchopulmonary foregut malformations. These lesions include congenital cystic adenomatoid malformations (CCAMs), sequestrations and infantile lobar emphysema. All congenital malformations of the lower respiratory tract are usually diagnosed and managed antenatally, in the newborn period, in infancy or in childhood. In a small number of patients, such malformations may go unrecognized in infancy, childhood and rarely in adulthood [1,2]. In the latter cases late complications, such as recurrent localized pneumonia, abscess formation, spontaneous pneumothorax, haemoptysis, or coincidental discovery on a chest radiograph may lead to the diagnosis. CCAM is a rare congenital pulmonary lesion, with a reported incidence of 1 in 25.000–35.000 pregnancies [3], involving maldevelopment of terminal branches, as a consequence of abnormal embryogenesis during the first 6–7 weeks of pregnancy [4,5]. It comprises a heterogeneous group of cystic and non-cystic lung lesions classified into three types by Stocker et al. in 1977 [4] on the basis of cyst size and macroscopic appearance. A late identification in adults is a rare event and previously reported cases almost always describe multiloculated cystic lesions.

We report a case of type I CCAM presenting as a single, expansive cystic mass in the middle pulmonary lobe in a 38-year-old man.

## Case presentation

**Figure 1:**
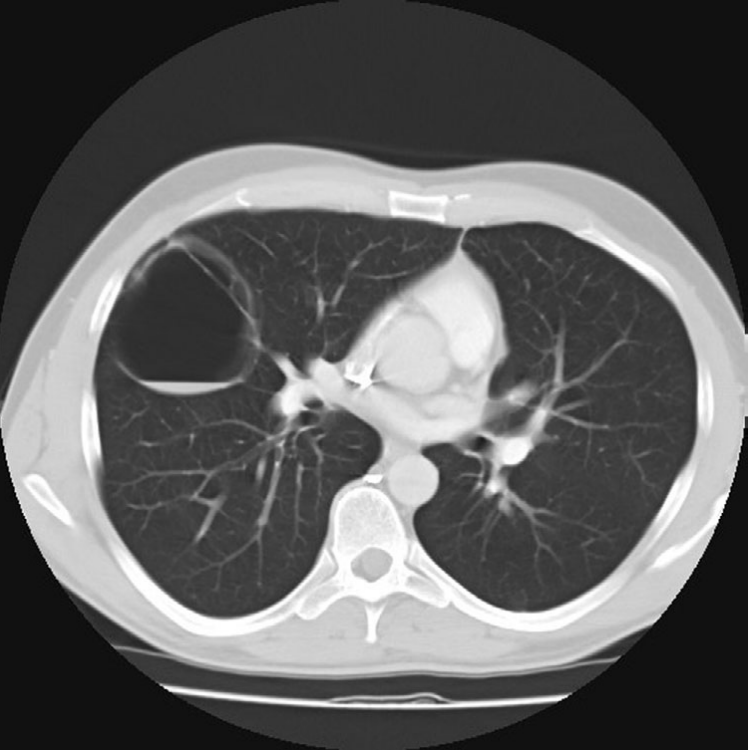
Contrast – enhanced computerized tomography of the chest. The right pulmonary lobe shows a uniloculated cyst with air fluid level.

A 38-year-old man was admitted to the Division of Surgery in February 2006 because of persistent cough and haemoptysis. Bronchoscopic examination, bronchoalveolar lavage, sputum and bronchial aspirate were negative for malignancies. Contrast-enhanced computed tomography scan disclosed a single expansive cystic mass, 7 cm in diameter, with air fluid level, occupying the lateral segment of the right middle lobe of the lung with compression of the medial segment and of the adjacent segments of the lower lobe. The cystic wall showed intraluminal projections and a sessile nodule 1 cm in diameter (Fig 1).

**Figure 2:**
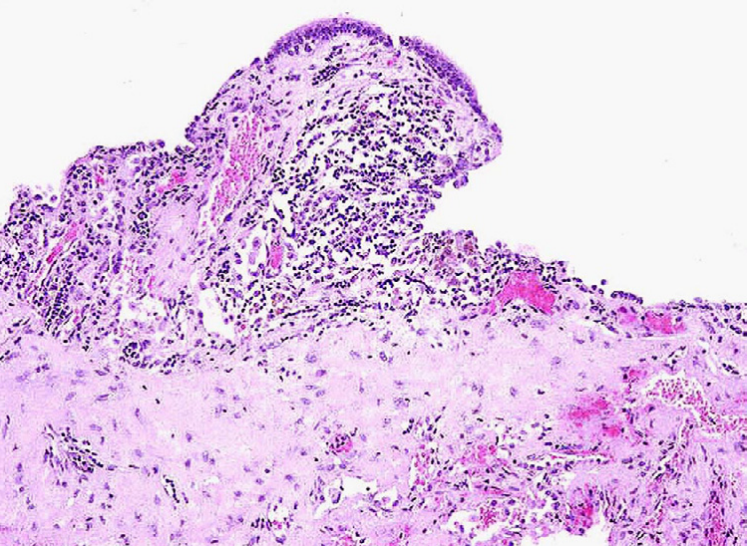
Congenital cystic adenomatoid malformation. The wall of the cyst is lined by cuboidal and pseudostratified, respiratory-like epithelium (hematoxylin and eosin, 200×).

**Figure 3:**
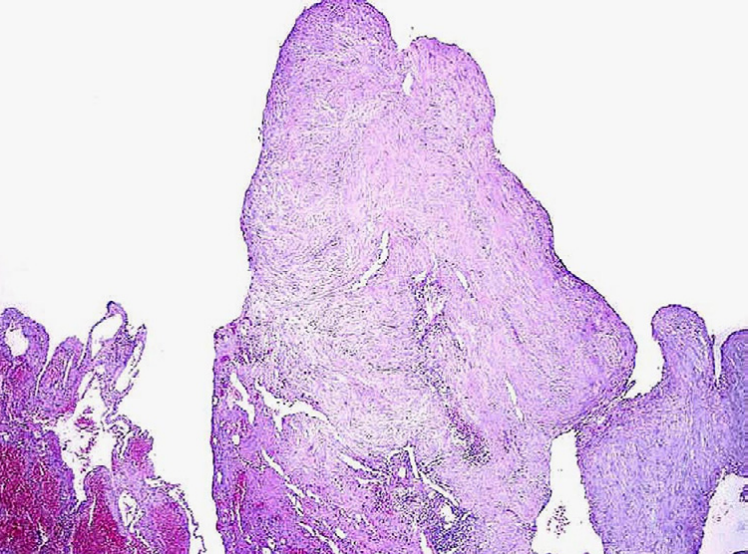
Congenital cystic adenomatoid malformation. Intraluminal projection of the cyst, made of fibrous connective tissue (hematoxylin and eosin, 200×).

The patient underwent a middle right lobectomy without complications. The macroscopic examination of the surgical specimen revealed a 7 × 6.5 × 6 cm single cyst, surrounded by haemorragic pulmonary tissue. The cavity contained 10 cc of clear fluid. The outer surface of the cyst was smooth and the inner surface was characterized by small papillary protrusions. Histologically, the cystic wall consisted of vascularized fibrous tissue lined by cuboidal or columnar respiratory epithelium with focal gland-like appearance (Fig 2). Rare smooth muscle bundles and elastic fibers were present. Islets of cartilage were not found. Intraluminal projections of connective tissue were observed (Fig. 3). The adjacent pulmonary parenchyma revealed areas of atelectasia and intra-alveolar essudate.

The diagnosis was CCAM, type I, according to the modified Stocker's classification [4].

The patient was discharged from the hospital one week later with a completely uneventful post operative course. Nine months after surgery the patient was free from symptoms.

CCAM is a relatively rare malformation of the terminal respiratory structures, first reported by Ch'in and Tang in 1949 [6]. The lesion consists of cysts and solid airless tissue, with no cartilage in the wall. It may affect partially or entirely the pulmonary lobes [7]. Many lesions described in the past as congenital cystic disease, bronchiolectasis, and a variety of related entities probably were adenomatoid malformations [8,9].

CCAMs have been classified, depending on the size and number of the cysts, into three types:

1. Type I (macrocystic type) accounts for 50% to 70% of cases, is characterized by multiple large cysts, (up to 10 cm) or a single dominate often multiloculated cyst, with a pseudostratified ciliated columnar epithelium, resembling the distal bronchial tree and proximal acinus, with normal alveoli between the cysts. Radiographic analysis may preoperatively suggest the diagnosis, especially when a multicystic pattern is evident. When the cystic lesion is single, the differential diagnosis with the congenital parenchymal cysts and bronchogenic cysts is not possible only on the base of the radiological features;

2. Type II (microcystic type) represents 20% to 40% of cases and shows multiple tiny cystic structures usually much less than 2 cm in diameter. This type can associate with high frequency to other congenital anomalies, and the prognosis is poor;

3. Type III (solid type) represents more than 10% of cases and consists of a bulky firm, solid mass with cysts less than 0.5 cm in diameter, mimicking the terminal bronchioles and the alveolar ducts of the pseudoglandular period[4,10,11]; it is now considered a form of pulmonary hyperplasia. The prognosis is usually poor.

CCAM is most commonly found in the neonatal period and up to 90% of diagnoses are made within the first two years of life [12,13].

The adult form of CCAM shows a wide radiological expression with extreme difficulty of preoperative diagnosis [14]. Some authors reported very unusual findings like the involvement of an entire lung lobe [15] or the presentation as a single large well defined cyst of more than 5 cm in diameter [16]. The disease may be asymptomatic, diagnosed by means of a routine chest radiograph [17], may be a surgical chance finding in the study of an extrapulmonary disease [16] or may be revealed by a lung inflammatory process [18,19]. The histological description lacks in almost all the reported cases in the literature, and the lesion is referred only to Stocker's classification. These previously post-natal and adult CCAM reported cases are enlisted in Table 1.

**Table 1:** Post-natal and adult CCAM cases. Review of the literature.

**Author**	**Cases**	**Age (years)**	**Sex**	**Clinical Findings**	**Site**	**Stocker's Classification**	**Treatment**
**Hellmuth**	23	from 18 to 65	11 M 12 F	8:recurrent pneumonia 5:asymptomatic 4:pneumothorax 3:haemoptysis 1:fever 39°C 1:multiple air-fluids levels on CT 1:dyspnoea	11:LLL 6:RLL 1:RUL 1:right lung base 1:ML 1:RMLL 1:bilateral involvement 1:not specified	19:I 3:II 1:not defined	16:lobectomy 2:cyst resection 1:right pneumonectomy 4:not described
**Dahabreh**	1	21	M	no symptoms	Posterior segment of RLL	I	Lobectomy
**Lujàn**	12	from 6 months to 23 years	8 M 4 F	9:recurrent pneumonia 2:chance finding 1:pneumothorax	3:LLL 6:RLL 2:LUL 1:RUL	7:I 4:II	8:lobectomy 2:segmentectomy 1:localized resection 1:waiting for surgical treatment at the time of publication
**Herrero**	2	46 47	1 M 1 F	1:pneumonia 1:asymptomatic	1:LLL 1:right parahilar mass	2:I	1:lobectomy 1:surgical resection
**Oh**	7	from 17 to 64	2 M 5 F	5:productive cough 1:haemoptysis 1:fever	2:LLL 1:LUL 2:RUL 1:RML 1:RLL	3:I 4:II	6:lobectomy 1:wedge resection

**Legend: **M = male; F = female; LLL = left lower lobe; LUL = left upper lobe; RLL = right lower lobe; RUL = right upper lobe; ML = middle lobe; RMLL = right middle and lower lobe.

## Conclusion

After a complete revision of the 45 cases of post-natal and adult CCAM reported in the literature, we can make the following considerations:

1. Type I is the most frequent CCAM type, representing the 64% of the described cases;

2. Only once a single, unilocular cystic mass was reported as the unique clinico-pathological manifestation in the lower right lobe [16];

3. CT scan provides a morphological assessment of the lung cavities, but it is inadequate to differentiate CCAM from other cystic lung diseases with similar imaging features. The differential diagnosis is essential, since malignancy has been associated with large cyst-type CCAM, including rhabdomyosarcoma [20] and bronchioloalveolar carcinoma [21]. This association was not found in others cystic lung diseases, particularly in the simple lung cyst.

When CCAM type I consists of a single large cyst, the differential diagnosis includes lung and bronchogenic cysts. The exact localization of the disease and the histological examination can be crucial for the correct diagnosis. Bronchogenic cysts are generally extrapulmonary, usually located in the right paratracheal or carenal region and may cause symptoms by bronchial compression or when infected. The histology of bronchogenic and lung cysts shows a columnar to cuboidal respiratory epithelial lining, surrounded by a fibromuscular wall which contains islands of cartilage and nests of bronchial glands. The single cyst of CCAM type I is always intrapulmonary and the wall is free of cartilage. The pathogenesis of the lesion is unknown. The absence of bronchiolar cartilage in the cystic wall suggests that in the type I there is an embryological alteration before the sixteenth week of intrauterine life, when the cartilaginous bronchi are formed. The histological picture of the present case supports this hypothesis.

In conclusion, the literature data confirm that surgical resection is the treatment of choice in all cases of CCAM and in the cases of cystic pulmonary lesions with uncertain radiological findings, in order to perform a histological examination of the lesion and to prevent infection and the potential neoplastic transformation [20,21].

## Competing interests

The author(s) declare that they have no competing interests.

## Authors' contributions

LM, IP, SL, SD, AC and FDN participated equally in the design of the report and in drafting the manuscript. All authors read and approved the final manuscript.
